# Biofortification of Chicken Eggs with Vitamin K—Nutritional and Quality Improvements

**DOI:** 10.3390/foods9111619

**Published:** 2020-11-06

**Authors:** Siobhan M. O’Sullivan, M. Elizabeth E. Ball, Emma McDonald, George L. J. Hull, Martin Danaher, Kevin D. Cashman

**Affiliations:** 1 Cork Centre for Vitamin D and Nutrition Research, School of Food and Nutritional Sciences, University College Cork, Cork T12 Y337, Ireland; siobhanosullivan@ucc.ie; 2 Agri-Food and Biosciences Institute, Veterinary Sciences Division, Stoney Road, Stormont Belfast BT4 3SD, Northern Ireland, UK; elizabeth.ball@afbini.gov.uk; 3 Devenish Nutrition Ltd., Belfast BT1 3BG, Northern Ireland, UK; emma.mcdonald@devenish.com; 4 Food Safety Department, Teagasc Food Research Centre, Ashtown, Dublin D15 DY 05, Ireland; george.hull@teagasc.ie (G.L.J.H.); martin.danaher@teagasc.ie (M.D.)

**Keywords:** egg, hen, vitamin K, menaquinone 4, MK-4, biofortification

## Abstract

National nutrition surveys have shown that over half of all adults in Ireland, the United Kingdom (UK), and the United States of America (USA) have low vitamin K intakes. Thus, dietary strategies to improve vitamin K intakes are needed, and vitamin K biofortification of food may be one food-based approach. The primary aim of our study was to establish whether increasing the vitamin K_3_ content of hen feed can increase the vitamin K content of eggs, and the secondary aims were to examine the effects on hen performance parameters, as well as egg and eggshell quality parameters. A 12 week hen feeding trial was conducted in which Hyline chickens were randomized into four treatment groups (*n* = 32/group) and fed diets containing vitamin K_3_ (as menadione nicotinamide bisulfite) at 3 (control), 12.9, 23.7, and 45.7 mg/kg feed. Vitamin K_1_, menaquinone (MK)-4, MK-7, and MK-9 were measured in raw whole eggs via a liquid chromatography tandem mass spectrometry method. MK-4 was the most abundant form of vitamin K (91–98%) found in all eggs. Increasing the vitamin K_3_ content of hen feed over the control level significantly (*p* < 0.001) enhanced the MK-4 content of eggs (mean range: 46–51 µg/100 g, representing ~42–56% of US Adequate Intake values). Vitamin K biofortification also led to significant (*p* < 0.05) increases in the yellowness of egg yolk and in eggshell weight and thickness, but no other changes in egg quality or hen performance parameters. In conclusion, high-quality vitamin K-biofortified eggs can be produced with at least double the total vitamin K content compared to that in commercially available eggs.

## 1. Introduction

Vitamin K has important physiological functions which relate to blood coagulation (its classical role), bone turnover and strength, inhibition of arterial calcification, and anti-inflammatory effects [1,2]. This relates to vitamin K’s molecular function in terms of serving as a cofactor for the enzyme γ-glutamate carboxylase, which catalyzes the conversion of glutamate into γ-carboxylated glutamate (Gla) in at least 17 different proteins distributed across biology and facilitating their full activity [3]. National nutrition surveys in Ireland (2008–2010) and the United Kingdom (UK) (2000–2001) have shown that 55% and 59% of nationally representative samples of the adult population had vitamin K_1_ intakes below the 1 µg/kg body weight required for coagulation [4,5]. Furthermore, the data shows that only about 19% and 34% of Irish men and women, respectively, and about 15% and 28% of men and women in the UK, respectively, met or exceeded the current sex-specific United States (US) Adequate Intakes (AIs) for vitamin K (120 and 90 µg/d, respectively [1]). In the US, 34% of adults aged ≥ 19 years had vitamin K_1_ intakes greater than the sex-specific AIs [6]. A triage theory suggests that, during poor dietary supply, vitamins are preferentially utilized for functions that are important to immediate survival [7]. This may explain why, in the healthy population, all clotting factors are synthesized in their active form, but the synthesis of other Gla-proteins is suboptimal [2]. Such prolonged subclinical vitamin K deficiency is considered a risk factor for osteoporosis, atherosclerosis, and cancer [8]. Of note, adults in the Irish national nutrition survey had, on average, ~40% of their serum osteocalcin in an undercarboxylated state (a widely used vitamin K status marker) [4].

The low habitual dietary intakes of vitamin K, as well as the fact that under-carboxylation of key Gla-proteins is common in the general population internationally [1,8], suggests that increased vitamin K intake is important in improving public health [2]. Clearly, while strategies are needed to improve vitamin K intakes, there are some important hurdles which point toward the need for additional creative food-based solutions. Vegetables and vegetable dishes are key food contributors to vitamin K intake [4,5,9]. Promoting greater vegetable consumption in the population, across all age groups, has been a mainstay of dietary guidelines over several decades, but adherence and uptake of this advice have been challenging [10,11]. Biofortification of foods with vitamin K could be an important potential complementary food-based approach to addressing low vitamin K intakes in the overall population, young and old. The concept of biofortification of foods with vitamins is gaining increasing attention internationally [12]. Eggs, ~6000 tons of which are consumed annually in Europe alone [13], have been shown to be an ideal vehicle for biofortication with vitamin D [14,15] and vitamin A [16,17]. Suzuki and Okamoto [18] showed how increasing the level of vitamin K_1_ or menadione (K_3_) in hen feed increased the vitamin K_1_ and/or menaquinone 4 (MK-4) content of eggs in a relatively short-term feeding trial (31 days). It should be noted, however, that the vitamin K_3_ content of feeds was increased 100- to 1000-fold over control level, and, other than the assessment of the number of eggs produced, no other hen performance or egg/eggshell quality parameters were assessed. Park et al. [19] in a 30 day hen feeding trial increased the vitamin K_3_ of hen feed in a dose-related manner (from 0.5 to 12.5 mg/kg diet) and investigated the effect on various hen performance and egg quality parameters, in addition to egg vitamin K content, but the vitamin D_3_ and iron content of the diet was also concomitantly increased [19]. It should also be noted that total vitamin K content of the eggs was assessed, which could not be delineated into vitamin K_1_ and menaquinones.

The aims of this study were to (1) establish whether increasing the vitamin K_3_ content of hen feeds, at approximately 4, 8, and 16 times the usual commercial level of addition, can increase the total vitamin K (i.e., vitamin K_1_ plus selected menaquinones) content of eggs (primary outcomes), and (2) to examine the effects on hen performance parameters, as well as egg and eggshell quality parameters (secondary outcomes).

## 2. Materials and Methods

All experimental procedures for the present hen feeding trial were approved under the Animal Welfare and Ethical Review Body at the Veterinary Sciences Division of the Agri-Food and Biosciences Institute (AFBI, Belfast, Northern Ireland) and were cognizant of the 3Rs principles in experimental animal research. All reagents were from Sigma-Aldrich (Wicklow, Ireland), unless otherwise stated.

### 2.1. Trial Design and Animal Management

A 12 week hen feeding trial was carried out within the Agriculture Branch of AFBI between May and August 2018. The trial was of a randomized design to test for the effect of treatment versus control and for the effect of increasing level of vitamin K_3_. Brown Hyline hens (*n* = 128), with a mean (± SD) initial body weight of 1.90 ± 0.03 kg, were purchased from Skea Eggs (Tyrone, Northern Ireland) and underwent an initial acclimatization period, where all hens received standard commercial feed for 4 days and a control diet (referred to as treatment 1, see below) for a further 5 days. Following this, the hens were randomly assigned to one of four dietary treatments on the basis of pen number with each group consisting of eight pens and four hens per pen. The desired levels of vitamin K_3_ addition to the feed were 3 mg/kg (which is the commercial norm and ensures the recommended allowance is met while also taking consideration of reduced stability) and then three higher test levels of addition at 12.5, 25, and 50 mg/kg diet. The following levels of vitamin K_3_ addition were those actually achieved at the feed manufacture stage, 3, 12.9, 23.7, and 45.7 mg vitamin K_3_/kg of feed, and these treatment diets were designated control, T1, T2, T3, and T4, respectively. The vitamin K_3_ was in the menadione nicotinamide bisulfite (MNB) form (Orffa Additives B.V., Werkendam, The Netherlands). The basic diet contained 200 g/kg crude protein, and all diets were formulated to meet nutrient requirements of the hen. Diets were kindly supplied by Devenish Nutrition Ltd. (Belfast, Northern Ireland). The diet was fed in pellet form, and each diet was made in 500 kg batches at the beginning of the experiment. Detailed ingredient composition and chemical analysis of the diets are presented in Table 1 and Table 2, respectively.

Hens were 22 weeks of age at the beginning of the trial, with 100% laying. Hens were provided feed ad libitum via circular feeders (Wells Poultry Equipment, Gwent, Wales, UK) and feed intake was recorded weekly by weighing feed used throughout the week. Water was also provided ad libitum through circular drinkers (Wells Poultry Equipment), and all treatment groups received a 14 h lighting pattern. Each pen was 61 cm in width, 150 cm in length, and 61 cm in height. Wood shavings (5 cm depth) were provided as bedding and scratching material, and nest areas were made in one corner of the pen with straw.

Eggs were collected daily, weighed, and recorded for each pen. Total egg production on a per pen and per hen basis was recorded, and percentage egg production calculated as total egg per hen divided by 84 × 100. Hen weight was also assessed at the end of the 12 week trial. Feed conversation–egg weight ratio (FCR) was calculated as feed intake divided by egg weight for the 12 week period.

### 2.2. Egg and Eggshell Quality Analysis

Egg quality was assessed at weeks 0, 4, 6, 8, and 12 of the trial. At each time point, eggs were collected and stored at 4 °C until shipping to University College Cork (UCC). Upon arrival in UCC, eggs were stored at 4 °C and analyzed within 14 days of being laid. Cracked and double-yolked eggs were excluded from the analysis. Egg quality parameters, such as Haugh unit (HU), yolk height, albumen height, and yolk color, as well as eggshell thickness and eggshell weight, were measured at each time point. Egg quality parameters were determined by weighing each egg, breaking it onto a flat glass top surface, and measuring the yolk and albumen height using electronic calipers (Technical Services and Supplies Ltd., York, England). The HU was determined using the following formula: HU = 100 log(H − 1.7W^0.37^ + 7.6), where H indicates the albumen height and W indicates the egg weight. Eggshell thickness was measured using a digital micrometer (Mitutoyo, Kawasaki, Japan). Eggs and eggshells were then packaged into airtight containers (Sarstedt, Nümbrecht, Germany) and stored at −20 °C until further analysis. Albumen weight, yolk weight, and antioxidant activity were also measured on a subset of eggs at each time point.

For eggshell calcium and magnesium analysis, eggshells were defrosted, and membranes were removed and washed using distilled deionized water. Samples were dried in an oven at 100 °C for 4 h and ground to a powder using a mortar and pestle. Samples (0.1 g) were then ashed in crucibles using a Bunsen burner and heated in a muffle furnace at 800 °C for 4 h. Samples were reweighed, dissolved in 25% HCl (Aristar grade) containing several drops of HNO_3_, and boiled. Samples were diluted as required for each mineral, and concentrations were determined using an atomic absorption spectrophotometer (Spectr AA-600; Varian Australia Ltd., Victoria, Australia). For the egg and eggshell quality parameters, priority was placed on the eggs collected from week 12 (endpoint).

### 2.3. Proximate Analysis of Experimental Diets and Eggs

Feed samples were dried at 55 °C for 72 h and then ground and sieved to remove large particles before proximate analysis. Whole-egg (yolk and albumen) samples from each treatment group (*n* = 3) were mixed using a blender and dried at 55 °C for 72 h before proximate analysis. Dried eggs were then ground using a mortar and pestle. Dry matter of feed and whole eggs was determined by oven drying at 100 °C for 17 h. Fat content of was determined using the CEM SMART System 5 rapid fat and moisture analyzer (CEM Corporation, Charlotte, NC, USA), whereas protein content was determined by the Kjeldahl method [20]. Ash content was determined by pre-ashing using a Bunsen burner and placing samples in a muffle furnace at 550 °C for 6 h.

### 2.4. Estimation of the Vitamin K_3_ Content of Experimental Diets and Eggs

Feed samples were also taken at several points during the 12 week trial and analyzed for menadione content to ensure levels remained stable. The menadione content of whole eggs was also measured. Menadione was extracted using the method described by Graff et al. [21], and its content determined by reversed-phase HPLC (High Performance Liquid Chromatography) with fluorescence detection.

### 2.5. Measurement of the Vitamin K_1_ and MK in Whole Eggs

The vitamin K_1_, MK-4, MK-7, and MK-9 in whole raw egg samples from the four treatment groups (*n* = 10/group) collected from weeks 0, 4, 8, and 12 were first extracted using an in-house sample preparation protocol (Food Safety Department, Teagasc Food Research Centre, Ashtown, Dublin, Ireland), which was based on liquid–liquid extraction following a lipase digestion. LC–MS/MS (Liquid Chromatography tandem Mass Spectrometry (HPLC)) analysis was performed on an Agilent Infinity™ II UHPLC (Agilent Technologies, Santa Clara, CA, USA) linked to an AB Sciex QTRAP 6500+ mass spectrometer (Sciex, Foster City, CA, USA) using atmospheric pressure chemical ionization (APCI) in the positive mode.

### 2.6. Antioxidant Activity of Egg Samples

Antioxidant activity was determined on 10 g of raw whole egg using a modified version of the method described by Duffy et al. [15]. After freeze-drying, samples were dissolved in 2 mL of methanol and filtered using a 0.22 µm syringe filter unit (Merck Millipore Ltd., Cork, Ireland). Antioxidant activity was determined using the 2,2-diphenyl-1-picrylhydrazyl (DPPH), total phenolic content (TPC), and the ferric reducing antioxidant power (FRAP) assays using methods described in detail elsewhere [22,23,24]. TPC and FRAP values are expressed as mg gallic acid equivalents/egg or µM FRAP equivalents/egg, respectively. DPPH values were expressed as mM Trolox equivalents/egg.

### 2.7. Statistical Analysis

Data were analyzed using Genstat Version 16.0 (VSNI, Hempstead, England, UK) and IBM SPSS Statistics for Windows, version 23 (IBM Corp., Armonk, NY, USA). The distributions of all variables were tested with Kolmogorov–Smirnov tests and any non-normally distributed variables were transformed, if possible, in an attempt to improve their normality before means testing. For egg and eggshell data, which were normally distributed, statistical differences between the treatment groups were determined using ANOVA followed by Bonferroni’s test. In the case where data transformation did not allow for more near-normally distributed variables, the Kruskal–Wallis tests followed by Mann–Whitney test for nonparametric data was used. For analysis of hen performance parameters, the pen was taken as the experimental unit and the effect of treatment was assessed using ANOVA. All tests were considered significant at *p* < 0.05. Data are expressed as means and their pooled standard errors (SEM).

## 3. Results

### 3.1. Effect of Dietary Vitamin K_3_ Treatment on Hen Performance Parameters

The effect of dietary vitamin K_3_ treatment on hen final body weight, feed intake, FCR, egg weight (average and total per group), number of eggs per hen, total number of eggs per pen, and percentage egg production are shown in Table 3. There was no significant difference across the four vitamin K_3_ dietary treatment groups in hen final body weight, feed intake, or FCR (*p* > 0.3, for all). Likewise, there was no significant difference across the four treatment groups in total egg weight or average egg weight (*p* > 0.18, for both). There was a nonsignificant trend (*p* = 0.08, for all) of a treatment effect on total number of eggs per pen, total eggs per hen, and percentage egg production, with the highest vitamin K_3_ treatment group (45.7 mg/kg diet) having modestly lower values (for example, 95% of that of the control group for each of the three parameters). 

### 3.2. Menadione Content of Diets and Eggs

The menadione content of each diet was measured and was found to remain stable during the trial. Increasing the level of vitamin K_3_ in feed did not increase the whole-egg menadione content; levels were below the level of detection across all treatment groups.

### 3.3. Effect of Dietary Vitamin K_3_ Treatment on Egg Vitamin K Content

The effect of dietary vitamin K_3_ treatment on mean vitamin K_1_, MK-4, and MK-7, as well as on estimates of total vitamin K content, in raw whole eggs at week 12 (endpoint) is shown in Table 4. Increasing the vitamin K_3_ content of the hen feed over the usual level of addition (3 mg vitamin K_3_/kg diet) more than doubled (*p* < 0.001) the MK-4 content of the eggs, with no significant differences among the three supplemental levels of addition (i.e., 12.9, 23.7, and 45.7 mg vitamin K_3_/kg diet) in egg MK-4 content achieved following 12 weeks of feeding. There was no significant difference (*p* > 0.4) in egg MK-7 content across the four dietary treatment groups, and egg vitamin K_1_ content significantly (*p* < 0.001) decreased with increasing vitamin K_3_ content of the hen feed. Vitamin K_1_ and MK-7 were only minor contributors to total vitamin content of the egg, with MK-4 contributing between 91% and 98%, depending on the vitamin K_3_ dietary level. The MK-9 content of egg was found to be below the limit of detection in all cases.

As a pilot study, two eggs from the 45.7 mg vitamin K_3_/kg diet group were cooked by poaching, and the MK-4 content was compared to that in two uncooked eggs (mean ± SD: 54.4 ± 0.2 versus 52.5 ± 0.3 µg/100 g, respectively).

**Figure 1:**
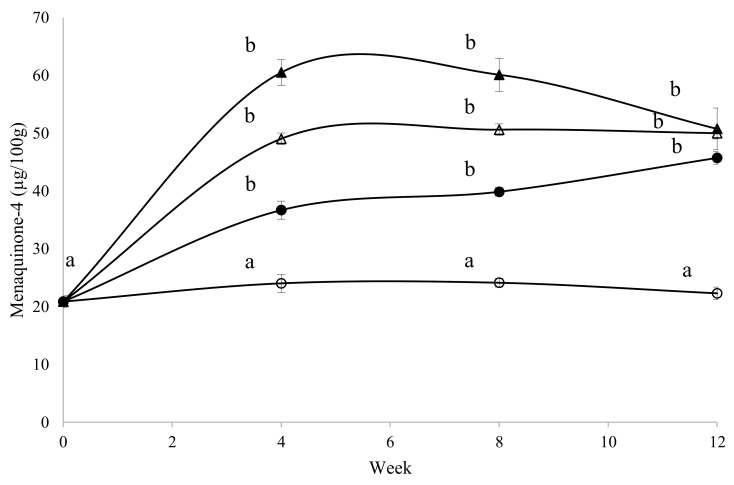
The mean menaquinone-4 content of whole eggs over the 12 weeks of the hen feeding trial, by dietary vitamin K_3_ treatment group (○, 3 mg of vitamin K_3_/kg of feed (control); ●, 12.9 mg of vitamin K_3_/kg of feed; ▲, 23.7 mg of vitamin K_3_/kg of feed; △, 45.7 mg of vitamin K_3_/kg of feed). Significance was determined using one-way ANOVA followed by Bonferroni’s post hoc tests. Values are means and standard errors for *n* = 10 eggs per group. Different letters represent statistical significance.

The effect of dietary vitamin K_3_ treatment on mean MK-4 content in whole eggs over the course of the trial is shown in Figure 1. Mean whole-egg MK-4 content in all three supplemental vitamin K_3_ treatment groups increased significantly at week 4 compared to baseline values (*p* < 0.001) and appeared to reach a plateau at this stage within the 12 week trial.

### 3.4. Effect of Dietary Vitamin K_3_ Treatment on Egg and Eggshell Quality Parameters

The effect of dietary vitamin K_3_ treatment on egg and eggshell quality parameters at endpoint are shown in Table 5. In terms of eggs, there was no significant difference across the four treatment groups in weight, HU, albumen height, yolk height, percentage yolk, or yolk-to-albumen ratio (*p* > 0.29, for all). Likewise, in relation to egg yolk color, there was no significant difference across the four treatment groups in L* (lightness) or A* (redness) of the egg yolk (*p* > 0.17, for both); however, there was a significant (*p* < 0.001) effect of dietary vitamin K_3_ treatment on b* (yellowness). Post hoc testing showed that b* was significantly higher in the three vitamin K_3_ dietary treatment groups compared to the control group, with the group receiving 12.9 mg vitamin K_3_/kg diet having the highest b* value (Table 5). There was no effect (*p* > 0.16, for all) of dietary vitamin K_3_ treatment on dry matter, ash, crude protein, or fat content of the eggs (Table 6).

In terms of eggshells, there were significant (*p* < 0.001, for both) effects of level of dietary vitamin K_3_ treatment on eggshell weight and thickness, with the groups receiving 12.9 and 23.7 mg vitamin K_3_/kg diet having higher mean eggshell weight and thickness compared to either control or the 45.7 mg vitamin K_3_/kg diet groups. While there was no effect (*p* > 0.8) of dietary vitamin K_3_ treatment on eggshell magnesium content, calcium content was modestly, but significantly (*p* < 0.05), higher in the group receiving 23.7 mg vitamin K_3_/kg diet compared to that in the group receiving 12.9 mg/kg diet, but there were no other differences between the groups (Table 5).

### 3.5. Effect of Dietary Vitamin K_3_ Treatment on Antioxidant Activity of Egg Samples

There was no significant difference across the four dietary vitamin K_3_ treatment groups in antioxidant activity of egg samples as assessed by the DPPH radical scavenging, TPC, and FRAP assays (*p* > 0.05, for all).

## 4. Discussion

The present hen feeding trial showed that the three supplemental levels of vitamin K_3_ (12.9, 23.7, and 45.7 mg/kg in the form of MNB) added to the hen feed were equally effective in increasing total vitamin K content of whole eggs (approximately twofold) over that achieved in eggs from hens fed the control diet containing the commercial level of vitamin K_3_ addition (3 mg/kg). The increase in vitamin K content of eggs was driven by an increase in MK-4 content, which was evident relatively quickly, and there was an apparent plateau in MK-4 content of eggs by week 4 within our 12 week trial. The MK-7 content of egg was unaffected, and the vitamin K_1_ content of the eggs actually decreased in a dose-dependent manner with increasing supplemental levels of vitamin K_3_. This decrease, however, was not of significant nutritional relevance as the level of vitamin K_1_ in eggs is naturally low to begin with (< 1 µg/100 g).

Suzuki and Okamoto [18] showed in a pilot trial (with only two eggs analyzed per dietary group and, thus, no statistical analysis) that, compared to a control diet (containing 1 mg vitamin K_3_/kg, and as menadione sodium bisulfite (MSB)), feeding diets containing 10, 20, and 50 mg vitamin K_3_/kg led to dramatically increased MK-4 content in egg yolks (~9-fold) over the 17 day trial period, but all to about a similar extent, whereas inclusion of 100 mg/kg increased the MK-4 content of the egg yolks further (a ~12-fold increment). In a second bigger trial within the same study, the authors compared the effect of supplying feeds containing 100, 200, 500, and 1000 mg vitamin K_3_/kg for 31 days on egg yolk vitamin K content. There was a dose-related increase in MK-4 content of the egg yolks (*n* = 7–9 eggs per group), and the MK-4 levels reached a plateau at 17 days after start of feeding the vitamin K_3_-enriched experimental feeds [18]. Using an assumption of 30% yolk within the eggs (as per the present study), the MK-4 content of eggs from the 50 mg vitamin K_3_/kg dietary group at the end of the 31-day hen trial by Suzuki and Okamoto [18] could be predicted to be ~40 µg/100 g whole egg, which is comparable to the 50 µg/100 g whole egg from hens receiving a similar dose of vitamin K_3_ at week 4 in the present study. Park et al. [19], also using a relatively short-term (30 day) hen feeding trial, tested the impact of inclusion of vitamin K_3_ (again as MSB) at 0.5 (control), 1.5, 5, 7.5, 10, and 12.5 mg/kg on the vitamin K content of eggs. Of note, however, they also concomitantly increased the vitamin D_3_ and iron levels in the feeds. The vitamin K content of the eggs reached a plateau at 20 days after the start of feeding the experimental feeds. The total vitamin K content of eggs collected during day 20 through 30 of the trial (as pooled samples of five eggs per diet per day) increased between 3.3- and 4.8-fold, depending on the feed vitamin K_3_ content, with the feed containing 7.5 mg/kg producing the greatest increase in total vitamin K content (53 µg/100 g whole egg) [19].

The findings of the vitamin K analysis of whole eggs from the hens fed the control diet in the present study show that the total vitamin K content (i.e., vitamin K_1_ plus MK-4 and MK-7) of 24.4 µg/100 g (of which only 0.9 µg/100 g was as vitamin K_1_) may be higher than that calculated from data in the UK food composition tables (at < 6 and 7.0 µg/100 g whole raw egg for vitamin K_1_ and K_2_, respectively, i.e., a total of < 13 µg/100 g maximum) [25]. The US Department of Agriculture’s (USDA’s) Food Data Central (FDC) food composition database reports a vitamin K_1_ content of 0.3 µg/100 g raw whole egg, but does not have an MK-4 content entry (only for scrambled egg (0.2 µg/100 g)) [26]. Elder et al. [27] using foods from the USDA’s Nutrient Data Laboratories, as part of the National Food and Nutrient Analysis Program, reported vitamin K_1_ and MK-4 contents of 0.3 and 5.6 µg/100 g fresh whole egg. The control diet in the present study contained 3 mg vitamin K_3_/kg feed according to the commercial level of addition, and a level set according to blood coagulation requirements, as well as consideration of reduced stability [28]. In contrast, the control diets used in the studies by Park et al. and Suzuki and Okamoto contained 0.5 and 1 mg vitamin K_3_/kg, respectively, leading to lower vitamin K contents of 11 and ~5 µg/100 g whole raw egg, respectively [18,19].

While vitamin K_1_ and MK-7 have been reported to possess good bioavailability [29], their contents in the eggs in the present study were relatively low at ≤ 1.2 µg/100 g egg. MK-4 has been shown to be bioavailable after oral ingestion in dogs and rats [30,31]. An increased intake of MK-4 has also been shown in several [32,33,34], but not all studies [35], to increase serum MK-4 levels in humans. These studies used supplemental MK-4 and, thus, the bioavailability of MK-4 from the vitamin K-biofortified eggs would need to be established. The oral bioavailability of MK-4 has been reported to be enhanced by increasing the fat content of a meal [36]; in our study, the eggs contained ~23% fat and, thus, may be an ideal vehicle to aid MK-4 bioavailability. Of note from a human nutrition perspective, a small pilot analysis in the present work also showed that cooking of the egg did not lead to any reduction in MK-4 content of the raw egg.

We chose to use MNB over MSB as the form of vitamin K_3_ to be added to the hen feeds. While both are regarded as effective sources of vitamin K in animal nutrition and are considered safe for all animal species at practical use levels in feed [37], MSB has been reported to induce toxicity in some animals [38] and to significantly reduce performance in fish [39]. In contrast, MNB has been shown to be well tolerated at high doses in fish and pigs [40,41]; as a result, MNB is the more commonly used form in animal nutrition. Importantly also, there is no maximum content limit for its use in animal nutrition [37]. In our 12 week feeding trial there was no overall effect of increasing vitamin K_3_ as MNB in the feeds on egg production parameters, although the highest supplemental level (45.7 mg vitamin K_3_/kg diet) led to a nonsignificant (*p* = 0.08) lower egg production (i.e., 5% less than that of the control group). It may be that, with a greater sample size, this decrease would have been significant and, at a commercial level, a confirmed 5% decrease in production would be of relevance. Suzuki and Okamoto [18] using a much higher dose range of vitamin K_3_ (1–1000 mg/kg diet), as MSB, reported no decrease in egg production in their 31 day trial. Importantly, they also found that menadione itself was nondetectable in the egg yolks [18], a finding also noted in the present study. Of note, the European Food Safety Authority’s Panel on Additives and Products or Substances Used in Animal Feed on reviewing the safety and efficacy of vitamin K_3_ in animal nutrition concluded that MNB does not give rise to safety concerns for consumers [37]. In the present study, there was no significant effect of the vitamin K_3_ treatment on other hen performance parameters such as hen weight, egg weight, feed intake, or feed conversion ratio.

In relation to egg quality parameters, there was no difference in ash, crude protein, or fat content of the eggs from hens fed the three supplemental levels of vitamin K_3_ addition to the feeds. Likewise, there was no effect of vitamin K_3_ treatment on antioxidant status of the eggs. Most non-nutritional egg quality parameters were unaffected by the level of vitamin K_3_ addition, except for yellowness of the egg yolk, which was significantly higher in eggs from the groups receiving the supplemental vitamin K_3_. Park et al. [19] also reported a tendency for egg yolk color index to be increased with increasing vitamin K_3_ and vitamin D_3_ content of the diets, but the effect of either vitamin on their own could not be delineated due to the formulation of the experimental diets. Yolk color, including the yellow-orange hue, is dependent on the hen’s diet, specifically, carotenoid intake [42]. The MNB used as the source of supplemental vitamin K_3_ in the present study is described by the manufacturer’s technical data sheet as yellowish in color and, thus, might explain an effect on egg yolk color. Surveys in European countries have confirmed that yolk color is one of the main parameters by which the quality of eggs is judged, and consumers prefer deeply hued yolks [43].

Park et al. [19] reported that feeding the hen diets containing 12.5 mg vitamin K_3_ (as MSB) per kg diet (but which also contained 20,000 International Units (IU) of vitamin D_3_/kg) led to a significantly higher proportion of eggs which were soft and broken compared to the other levels of addition. In the present study, the eggshells from hens fed the 12.9 mg and 23.7 mg, but not 45.7 mg, vitamin K_3_ (as MNB) per kg diet were significantly heavier and thicker than those from hens fed the control diet. A reduction in the number of breakages would contribute toward achievement of the United Nations 2030 Sustainable Development Goals target of a reduction in food losses at the consumer, retail, and reduction level [44]. The recent report from the EAT-Lancet Commission also highlighted the importance of reducing food loss during food production and set a goal to achieve a reduction in global food loss and waste of 50% [45].

## 5. Conclusions

Overall, the findings of the present 12 week hen feeding trial showed that vitamin K-biofortified eggs can be produced with at least double the total vitamin K content compared to that in eggs from conventional feed. This can be achieved without significant adverse effects on hen performance and with improvement in egg yolk yellowness, as well as increased eggshell weight and thickness. Including vitamin K_3_, as the MNB form, at the level of 25 mg/kg hen feed, which was shown to double the vitamin K content of the egg, increase eggshell weight and thickness, but have no effect on egg production, would translate into an additional cost in manufacture of approximately EUR 0.37 per ton of feed.

## Figures and Tables

**Table 1:** Composition of basal diet offered to hens over the 12 week feeding trial.

Ingredient	(g or IU */kg Feed)
Wheat	655
Hypro soya	218
Limestone flour/grit	85
Soya oil	22
Dicalcium phosphate	9.5
Sodium chloride	3.0
Sodium bicarbonate	2.0
Methionine	1.5
Lysine	0.5
Vitamins/minerals:	
Retinyl acetate *	8000
Cholecalciferol *	3000
Calcium carbonate	1.906
Manganese	0.145
Iron	0.100
Zinc	0.097
α-Tocopherol	0.060
Nicotinic acid	0.040
Copper	0.040
Cobalamin	0.020
Calcium-d-pantothenate	0.006
Selenium	0.004
Riboflavin	0.004
Menadione ^1^	0.003
Iodine	0.002
Thiamine	0.001
Pyroxidine	0.001
Folic Acid	0.0003
Color	0.925
Antioxidant	0.125
Xylanase	0.035
Phytase	0.025

^1^ Vitamin K_3_ (as menadione nicotinamide bisulfite) was added to basal diet at four levels: (T1) 3 mg of vitamin K_3_/kg of feed (control); (T2) 12.9 mg of vitamin K_3_/kg of feed; (T3) 23.7 mg of vitamin K_3_/kg of feed; (T4) 45.7 mg of vitamin K_3_/kg of feed. *Denotes where addition presented in International Units (IU) per kg feed.

**Table 2:** Analyzed select chemical profile of four experimental diets.

	Treatment 1	Treatment 2	Treatment 3	Treatment 4
Dry matter (g/kg)	892.9	889.9	873.4	868.3
Ash (g/kg)	69.8	107.2	94.5	73.7
Crude protein (N × 6.25) (g/kg)	199.6	193.3	197.5	196.3
Fat (g/kg)	27.8	20.8	16.7	16.9

**Table 3:** Effect of dietary vitamin K_3_ treatment (T) ^1^ on hen weight, feed intake, and egg production throughout the 12 weeks of the trial. SEM, standard error of the mean.

	T1 (*n* = 32)	T2 (*n* = 32)	T3 (*n* = 32)	T4 (*n* = 28)	Pooled SEM	*p*-Value *
Hen start weight (g)	1890	1897	1931	1904	23.0	0.614
Hen end weight (g)	1987	2066	2004	2011	30.2	0.297
Total egg weight (g)	20,168	20,311	19,982	19,306	338.6	0.185
Total number of eggs/pen	314.6	315.8	313	299.1	4.90	0.081
Average egg weight (g)	64.1	64.3	63.9	64.6	0.621	0.849
Feed intake (g/hen/day)	128.8	130.8	126.8	133.3	4.98	0.815
FCR (intake–egg weight)	2.14	2.17	2.13	2.32	0.084	0.362
Total eggs/hen	78.7	78.9	78.2	74.8	1.224	0.081
Egg production (%)	93.6	94.0	93.1	89.0	1.457	0.081

^1^ Details of the four dietary vitamin K_3_ treatments as per Table 1. * Via one-way ANOVA and using pen as the experimental unit. FCR, feed conversation–egg weight ratio.

**Table 4:** Effect of dietary vitamin K_3_ treatment (T) ^1^ on vitamin K_1_ and menaquinone content of whole eggs at trial endpoint.

	T1 ^1^	T2	T3	T4	Pooled SEM	*p*-Value
Phylloquinone (µg/100 g)	0.87 ^a^	0.76 ^ab^	0.51 ^bc^	0.38 ^c^	0.22	0.00 *
Menaquinone-4 (µg/100 g)	22.30 ^a^	45.73 ^b^	50.00 ^b^	50.74 ^b^	8.99	0.00 *
Menaquinone-7 (µg/100 g)	1.22	0.97	0.71	0.86	0.68 ^#^	0.45 ^Ψ^
Total vitamin K_2_ (µg/100 g) ^2^	23.52 ^a^	46.70 ^b^	50.71 ^b^	51.60 ^b^	9.30	0.00 *
Total vitamin K (µg/100 g) ^3^	24.39 ^a^	47.46 ^b^	51.22 ^b^	51.99 ^b^	9.35	0.00 *

^1^ Details of the four dietary vitamin K_3_ treatments as per Table 1. Values represent 10 eggs/treatment group. ^2^ Representing the sum of menaquinone-4 and menaquinone-7 content. ^3^ Representing the sum of phylloquinone, menaquinone-4, and menaquinone-7 content. * Via one-way ANOVA followed by Bonferroni’s post hoc tests with different superscript letters representing significant (*p* ≤ 0.01) differences among group means. ^Ψ^ Via Kruskal–Wallis followed by Mann–Whitney U test. ^#^ Pooled SEM for non-normally distributed data was calculated as per for normally distributed data.

**Table 5:** Effect of dietary vitamin K_3_ treatment (T) ^1^ on egg and eggshell quality parameters at trial endpoint.

	T1	T2	T3	T4	Pooled SEM	*p*-Value ^¶^
Egg:						
Weight (g)	63.5	67.4	63.9	65.1	5.32	0.289
Haugh unit	98.9	97.8	95.6	95.8	5.77	0.435
Albumen height (mm)	7.84	7.74	7.27	7.20	1.16	0.409
Yolk height (mm)	17.7	17.7	17.1	17.2	1.03	0.543
Percentage yolk (%)	29.9	28.6	30.3	30.3	2.47	0.600
Yolk/albumen ratio	0.52	0.51	0.53	0.53	0.055	0.842
Yolk color:						
L*	51.6	54.3	52.9	53.2	2.96	0.166
A*	−3.4	0.9	0.8	−0.9	0.21	0.274
b*	27.3 ^a^	39.6 ^b^	36.6 ^bc^	34.7 ^c^	3.44	0.000
Eggshell:						
Weight (g)	6.2 ^a^	7.9 ^b^	7.5 ^bc^	6.8 ^ac^	0.76	0.000
Thickness (mm)	0.28 ^a^	0.36 ^b^	0.36 ^b^	0.32 ^a^	0.03	0.000
Calcium content (%)	32.9 ^ab^	32.5 ^a^	35.7 ^b^	34.3 ^ab^	2.48	0.035
Magnesium content (%)	0.39	0.41	0.40	0.39	0.06	0.886

^1^ Details of the four dietary vitamin K_3_ treatments as per Table 1. Values represent 10–12 eggs/treatment group for all parameters except for % yolk and yolk/albumen ratio, which were the means of six eggs/treatment group. **^¶^** Via one-way ANOVA followed by Bonferroni’s post hoc tests with different superscript letters representing significant (*p* ≤ 0.01 for all, bar ≤ 0.05 for calcium content) differences among group means. L*, lightness; A*, redness; b*, yellowness.

**Table 6:** Effect of dietary vitamin K_3_ treatment (T) ^1^ on proximate analysis of whole eggs at trial endpoint.

	T1	T2	T3	T4	Pooled SEM	*p*-Value *
Dry matter (g/kg)	254.9	268.3	249.7	251.2	0.490	0.163
Ash (g/kg)	26.4	29.5	27.1	27.4	0.017	0.235
Crude protein (N × 6.25) (g/kg)	405.5	387.5	350.0	377.2	12.325	0.525
Fat (g/kg)	231.7	235.7	214.2	223.9	11.369	0.920

^1^ Details of the four dietary vitamin K_3_ treatments as per Table 1. Values represent three eggs/treatment group. * Via one-way ANOVA.
